# Implicit and explicit self-concepts of forgiveness in women with borderline personality disorder

**DOI:** 10.1186/s40479-025-00312-4

**Published:** 2025-09-02

**Authors:** Philipp Wülfing, Carsten Spitzer, Nikolaus Krämer, Emanuel Severus, Ramzi Fatfouta

**Affiliations:** 1 Department of Psychosomatic Medicine and Psychotherapy, University Medical Center Rostock, Rostock, Germany; 2 Asklepios Clinic North — Ochsenzoll, Hamburg, Germany; 3 Independent Researcher, Berlin, Germany

**Keywords:** Borderline personality disorder, Forgiveness, Interpersonal forgiveness, Implicit association test (IAT)

## Abstract

**Background:**

The tendency to forgive is associated with traits such as agreeableness and neuroticism, mental well-being, and interpersonal functioning. Given documented associations with interpersonal conflict and aggression in borderline personality disorder (BPD), forgiveness (or, lack thereof) may be particularly relevant for BPD symptomatology but remains understudied. This study examines forgiveness in BPD compared to a heterogeneous clinical control group without personality disorder (CC), exploring its associations with aggression and interpersonal dysfunction using both direct (self-reported) and indirect (implicit) measures.

**Methods:**

Fifty-one female BPD patients and fifty-one CC participants completed self-report measures of forgiveness (Transgression-Related Interpersonal Motivations Inventory, Tendency to Forgive Scale) and a Forgiveness Implicit Association Test (F-IAT), alongside assessments of borderline symptoms, aggression, and interpersonal problems. Independent-samples *t*-tests compared the two samples, while Pearson correlations explored associations between clinical characteristics within the BPD sample.

**Results:**

Both groups revealed largely comparable scores in both explicit and implicit forgiveness, with no significant differences across measures. Within the BPD group, higher TRIM–Revenge scores were associated with greater aggression, particularly anger, while TTF scores showed negative associations with overall aggression, physical aggression, and hostility. Circumplex analysis indicated that the F-IAT aligned with nonassertive, TRIM–Revenge with cold and competitive, and TTF with warm and non-dominant interpersonal styles, whereas TRIM–Avoidance and TRIM–Benevolence exhibited limited interpersonal relevance.

**Conclusions:**

Despite limited prior research suggesting reduced forgiveness in BPD, the present findings indicate that women with BPD exhibit forgiveness tendencies comparable to those of the CC. Notably, explicit forgiveness was systematically associated with aggression and interpersonal difficulties, whereas implicit and explicit measures showed limited convergence. These findings underscore the utility of a multidimensional approach to assessing forgiveness in BPD, revealing distinct clinical and interpersonal correlates across forgiveness dimensions.

**Supplementary Information:**

The online version contains supplementary material available at 10.1186/s40479-025-00312-4.

## Background

Dispositional forgiveness, also referred to as the tendency to forgive, can be conceived as a relatively stable personality trait that influences how readily an individual forgives others over time and across various interpersonal situations [1, 2]. It reflects a person’s general propensity to let go of resentment, avoid retaliation, and extend mercy or understanding toward others who have wronged them. Because high forgiveness repairs, maintains, and even improves relationships after transgressions have occurred, it has a prosocial interpersonal effect [3, 4]. Moreover, it also has a positive intrapersonal effect as it can help individuals overcome interpersonal violations, especially negative emotions such as anger, worry, fear, and embarrassment, reduce individual anxiety and depression, and improve self-esteem, subjective well-being [5] and mental health [6].

Several lines of research suggest that forgiveness may be clinically meaningful in borderline personality disorder (BPD), characterized by a pervasive pattern of instability in self-image, emotions, and interpersonal relationships [7–9]. First, within the Five Factor Model (FFM) of personality the empirically most replicated personality correlates of forgiveness are (high) agreeableness and (low) neuroticism, as supported by a systematic review [10] and meta-analysis [11]. The aggregate effect sizes for the associations of forgiveness with agreeableness and neuroticism are substantial (*r* =.44 and *r* = -.28, respectively) [11]. Notably, these traits are also meaningfully associated with BPD, where individuals typically exhibit lower levels of agreeableness and higher levels of neuroticism, thus supporting the relevance of forgiveness-related personality dimensions in the context of BPD [12].

Second, psychopathological features like depression, anxiety or other negative emotions which are often seen in BPD are inversely associated with forgiveness [13–15]. For example, “inappropriate, intense anger or difficulty in controlling anger” is one of the nine diagnostic criteria for BPD according to the DSM-5 [7], and meta-analytic evidence indicates a close association between forgiveness and state anger with a weighted mean correlation of *r* = -.41 [16]. Third, BPD is frequently associated with childhood maltreatment (CM) and early life adversity [17, 18], and several studies suggest a negative association between traumatic stress, including CM, and forgiveness [19, 20]. While BPD is often associated with interpersonal difficulties, studies have found that individuals with BPD show a 2.6 times higher risk of engaging in violent behavior compared to the general population [21, 22]. As interpersonal difficulties are a core feature of BPD [9, 23], it is plausible that low levels of forgiveness may hinder cooperative behavior and contribute to broader patterns of social dysfunction. Although forgiveness has been discussed in relation to personality traits, emotional dysregulation, and trauma [10, 11, 13, 14, 19, 20], its specific role in BPD has received limited empirical attention.

For example, a qualitative study [24] revealed that longing for reconciliation influences the daily lives of women with BPD and early adversity, as well as their search for the meaning of CM. Holm et al. suggest that forgiveness is key to developing an empathic understanding of oneself in the context of past and present relationships. Using the Forgiveness Scale [25] and the BPD Scale of the Personality Diagnostic Questionnaire-4 (PDQ-4) [26], a cross-sectional investigation in internal medicine outpatients indicated a negative and medium effect sized relationship between forgiveness and borderline pathology (*r* = -.55) [27]. Moreover, a large-scale web-based study focusing on cooperative behavior indicated that borderline pathology is related to impaired reactive cooperation, but unrelated to active cooperation, suggesting that individuals with high levels of BPD features have problems with forgiveness [28]. The McLean Study of Adult Development (MSAD) further revealed that BPD patients had markedly lower scores for indicators of forgiveness than comparison subjects with personality disorders other than BPD [29]. However, these scores were not static: forgiveness increased significantly over 20 years of prospective follow-up for the BPD group, but not for the control group. Additionally, recovered BPD patients reported approximately three times higher levels of forgiveness indicators than non-recovered patients at the end of the follow-up period [29]. Finally, a manualized group forgiveness module within dialectical behavior therapy (DBT) for BPD patients showed promising results: The intervention increased forgiveness and decreased attachment insecurity and psychiatric symptoms; these effects were maintained during the 6-week follow-up [30].

Despite the valuable contributions of the aforementioned studies, it has to be kept in mind that the Forgiveness Scale [25] used in the investigation of Sansone and co-workers [27] captures a variety of aspects of forgiveness, but not specifically the tendency to forgive. Moreover, this measure has not been psychometrically evaluated. Zanarini and colleagues [29] used two items from the Positive Affect Scale (PAS) as proxies for forgiveness (i.e., “accepting of the past” and “that I’ve forgiven those who’ve hurt me”), but no reliable and valid self-report instrument that specifically taps into dispositional forgiveness [10].

Although the use of well-validated questionnaires to measure forgiveness in individuals affected by BPD can be considered a step forward, their exclusive application might be problematic for several reasons. First, the reliance on one measurement mode can result in mono-method bias possibly increasing the probability of shared error variance in results [31]. Second, forgiveness represents a value-laden construct – while some view it as socially desirable, others view it as a sign of weakness and therefore undesirable [32]. As a result, self-report measures of forgiveness are particularly susceptible to self-presentation biases [33]. Third, they are insensitive to processes outside of awareness [34]. For example, one might explicitly state that one has forgiven an offense, yet implicitly continue harboring a grudge (hollow forgiveness [35, 36]). Thus, incorporating indirect measures in addition to direct ones may be useful to capture forgiveness on both explicit and implicit levels. To this end, we employed the Forgiveness Implicit Association Test (F-IAT) [37] as part of an exploratory, multimethod approach to capture forgiveness-related self-associations beyond self-report. As a latency-based categorization task, the F-IAT assesses the strength of automatic associations between the self (e.g., “me”) and forgiveness-related attributes (e.g., “forgiving” vs. “vengeful”) by measuring reaction times in a speeded classification paradigm. The core assumption is that individuals who implicitly associate themselves with forgiveness respond faster to congruent pairings (e.g., “me–forgiving”) than to incongruent ones (e.g., “me–vengeful”). The F-IAT has demonstrated good internal consistency and incremental validity in predicting forgiveness-related behavior beyond self-report, and may therefore help to circumvent common limitations of direct measures such as social desirability or limited introspective access [37].

In light of the above-mentioned findings and theoretical considerations, our exploratory study aimed to examine whether forgiveness differs between individuals with BPD and a clinical control group (CC) without BPD. By integrating both direct and indirect measures of forgiveness, we aimed to capture explicit and implicit forgiveness tendencies, thereby addressing potential self-presentation biases and unconscious processing of transgressions [31, 33, 37]. Given the high comorbidity of BPD with other mental disorders (e.g., mood, anxiety, somatoform, and eating disorders [9]), our matched CC consisted of patients diagnosed with these conditions. As previous research suggests gender differences in forgiveness tendencies (with women being more forgiving than men), we confined our sample to female patients to ensure homogeneity [38]. Building upon prior findings [27, 29], we further investigated the interrelations between forgiveness and aggression in BPD. Given the inherently relational nature of forgiveness, we also included a measure of interpersonal problems to examine how dispositional forgiveness may relate to individual patterns of interpersonal functioning. This approach aimed to situate forgiveness within the broader relational context that is often relevant in the clinical presentation of BPD.

## Methods

### Transparency and openness

The code for reproducing all analyses is available at https://osf.io/rba48/. For ethical reasons, the study data are available only upon reasonable request from the last author. This study and its analyses were not preregistered. In accordance with JARS guidelines [39], we report our sample size determination, all data exclusions, and all measures used. The research was approved by the Ethics Committee of the Medical Faculty at the University of Rostock (case number: A2021-0074) and conducted in accordance with the Declaration of Helsinki.

### Participants and data collection

This study used a sample of 51 female patients diagnosed with BPD and a CC of 51 female patients, matched for age and educational background. Participants were recruited through the Clinic for Psychosomatic Medicine and Psychotherapy at the University of Rostock. BPD diagnoses were established by experienced board-certified psychiatrists and confirmed using the German version of the Structured Clinical Interview for DSM-5 Personality Disorders (SCID-5-PD) [40], administered by the corresponding author. Additionally, all participants were screened for current mental disorders using the German version of the MINI-DIPS [41]. Exclusion criteria included age under 18, a body mass index (BMI) below 18 kg/m², neurological disorders, psychotic symptoms, mania, substance dependence or active substance use, and an estimated IQ below 70 (assessed via the German Multiple-Choice Vocabulary Test, MWT-B) [42]. All participants provided written informed consent. The initial screening included 119 individuals, of whom 11 were excluded during diagnostic assessment due to not meeting the inclusion criteria (see Figure S1).

### Measures

#### Assessment of forgiveness

##### Forgiveness Implicit Association Test (F-IAT)

The Implicit Association Test (IAT) [43] is a latency-based categorization task designed to measure the strength of associations between target concepts (e.g., “me” vs. “not me”) and specific attributes (e.g., “forgiving” vs. “vengeful”). Fatfouta and colleagues developed and validated a forgiveness-specific F-IAT [37], which was administered in this study using SoSciSurvey, a software platform for scientific surveys [44]. The F-IAT followed the standard seven-block structure: Blocks 1 and 2 introduced the target (me vs. not me) and attribute categories (“forgiving” vs. “vengeful”), while Blocks 3–4 and 6–7 presented the critical pairings in both initial and reversed configurations. We applied the improved scoring algorithm [45], which calculates the *D*-score by dividing the mean response time difference between critical blocks by the standard deviation of response latencies. Higher D-scores indicate stronger implicit forgiveness. The F-IAT exhibited good internal consistency, with a Spearman-Brown split-half reliability of 0.89 in the original study [37], and 0.87 in the BPD sample and 0.91 in the CC sample.

##### Transgression-Related Interpersonal Motivations Inventory (TRIM)

The TRIM [46] is a self-report measure that assesses motivational tendencies toward a specific transgressor across three subscales: Revenge (e.g., “I want him/her to get what he/she deserves”), Avoidance (e.g., “I would avoid him/her”), and Benevolence (e.g., “Even though his/her actions hurt me, I still have goodwill for him/her”). The TRIM consists of 18 items, rated on a five-point Likert scale (1 = *strongly disagree* to 5 = *strongly agree*), with higher scores indicating stronger endorsement of the respective motivational tendency. The scale has demonstrated high internal consistency in previous studies [46, 47]. In the present study, McDonald’s ω values were 0.83 (BPD) and 0.90 (CC) for Revenge, 0.90 (BPD) and 0.94 (CC) for Avoidance, and 0.93 (BPD) and 0.94 (CC) for Benevolence.

##### Tendency to Forgive Scale (TTF)

The TTF [1] is a brief self-report instrument designed to measure individual differences in the general propensity to forgive transgressions across different contexts and relationships. It comprises four items, each rated on a five-point Likert scale (1 = *strongly disagree* to 5 = *strongly agree*). The scale has demonstrated reasonable internal reliability and high test-retest stability over an eight-week period [1]. In the present study, McDonald’s ω for the Total score was 0.70 in the BPD sample and 0.60 in the CC sample.

##### Borderline Symptom List Short Version (BSL-23)

The BSL-23 [48] is a 23-item self-report measure assessing the severity of borderline symptoms over the past week. Items are rated on a five-point Likert scale (0 = *not at all* to 4 = *very strong*) and align with DSM-IV/DSM-5 diagnostic criteria. Since the short form does not assess self-harm behavior, we additionally administered the self-harm subscale from the BSL-95 [49]. The questionnaire demonstrated strong psychometric properties, with McDonald’s ω = 0.92 for the BPD sample and ω = 0.90 for the CC sample.

##### Aggression Questionnaire (AQ)

The AQ [50] is a 29-item self-report measure assessing dispositional tendencies toward anger and aggression across four subscales: Physical Aggression (e.g., “If somebody hits me, I hit back”), Verbal Aggression (e.g., “I often find myself disagreeing with people”), Anger (e.g., “I have trouble controlling my temper”), and Hostility (e.g., “I wonder why I sometimes feel so bitter about things”). Items are rated on a five-point Likert scale (1 = *extremely uncharacteristic of me* to 5 = *extremely characteristic of me*). McDonald’s ω for the total score was 0.85 in the BPD sample and 0.81 in the CC sample. Subscale reliability in the BPD sample ranged from 0.69 (Anger) to 0.85 (Physical Aggression), while in the CC sample, it ranged from 0.63 (Verbal Aggression) to 0.84 (Hostility).

##### Inventory of Interpersonal Problems-32 (IIP-32)

The IIP-32 [51] is a 32-item self-report measure assessing interpersonal difficulties. Items are rated on a five-point Likert scale (0 = *not at all* to 4 = *very much*), indicating the extent to which each statement applies to the respondent. Based on the interpersonal circumplex model [52], the items are grouped into eight octants along the Agency-Communion axes, which were used in this study. The scale demonstrated satisfactory psychometric properties, with McDonald’s ω for the total score at 0.80 in the BPD sample and 0.83 in the CC sample. Subscale reliability was also adequate, with ω values of 0.73 (Communion) and 0.79 (Agency) in the BPD sample, and 0.80 (Communion) and 0.81 (Agency) in the CC sample.

#### Data analysis

All statistical analyses were conducted using *R* (version 4.2.3) [53]. Chi-square tests were used to compare diagnostic frequencies between subsamples. Internal consistency of all measures was assessed using McDonald’s ω. Socio-demographic variables and primary outcomes were compared using Welch‘s *t*-tests for independent-samples with Bonferroni-Holm correction. Effect sizes for these comparisons are reported as Cohen’s *d* (*d* ≈ 0.2 = small, *d* ≈ 0.5 = medium, *d* ≈ 0.8 = large). Pearson correlation coefficients were computed for all measures. To further investigate the relationship between forgiveness and interpersonal dysfunction, we applied the Structural Summary Method (SSM) [54]. This method projects all forgiveness-related outcomes (F-IAT, TRIM, TTF) onto the circumplex structure defined by the IIP-32, modeling their association with interpersonal traits as a cosine curve. The SSM provides four key parameters to describe these associations: Elevation (e) reflects the general association of a construct with interpersonal dysfunction, with higher values indicating greater overall sensitivity to aversive interpersonal behaviors. Bootstrapped 95% confidence intervals (2,000 replicates) were used to determine whether elevation significantly differed from zero. Amplitude (α) represents interpersonal specificity, indicating how strongly a variable aligns with a distinct interpersonal style. Based on previous research [54], amplitudes of 0.10, 0.16, and 0.23 are considered small, average, and large effects, respectively. Angular displacement (δ) identifies the predominant interpersonal theme with which a construct is most aligned (e.g., intrusive, exploitable, competitive). Displacement was only interpreted for measures that exhibited a prototypical correlation pattern, meaning the pattern of correlations followed a cosine wave. Following recommendations, we set a model fit threshold of *R*² ≥ 0.70 for interpreting displacement [55, 56]. Model fit (*R*²) reflects how well the construct conforms to the circumplex structure, with values exceeding this threshold indicating that a construct’s circumplex projection is interpretable.

## Results

The BPD and CC samples were matched for age and years of education, with no significant differences between them. As expected, women with BPD scored significantly higher on measures of borderline pathology, including the BSL-23 total score and the number of BPD criteria, compared to the CC sample (Table 1). Additionally, BPD patients had a higher number of comorbid diagnoses, as assessed by the Mini-DIPS. A detailed breakdown of comorbidities is provided in the supplementary material (Table S1).

**Table 1:** Demographic and clinical characteristics of BPD (*N* = 51) and CC sample (*N* = 51)

	BPD	CC	Statistics		
	M ± SD	M ± SD	t	*p*	d
Age	33.27 (10.22)	35.76 (12.70)	1.09	0.834	0.22
Years of education	13.86 (2.15)	14.49 (1.92)	1.56	0.583	0.31
No. of BPD criteria (SCID-5-PD)	6.84 (1.72)	0.35 (0.87)	24.01	< 0.001	4.75
No. of diagnoses	4.02 (2.01)	1.88 (1.38)	6.27	< 0.001	1.24
Borderline pathology (BSL-23)	62.69 (17.81)	43.35 (11.87)	6.45	< 0.001	1.28
**Forgiveness (F-IAT**,** TRIM**,** TTF)**					
F-IAT	0.34 (0.31)	0.41 (0.29)	1.06	0.834	0.21
TRIM – Revenge	1.89 (0.81)	1.73 (0.77)	1.00	0.834	0.20
TRIM – Avoidance	3.19 (0.78)	2.94 (0.82)	1.58	0.583	0.31
TRIM – Benevolence	2.77 (0.72)	3.10 (0.75)	2.26	0.206	0.45
TTF	12.18 (4.26)	13.75 (4.06)	1.9	0.359	0.38
**Aggression (**AQ**)**	78.04 (13.57)	60.75 (10.97)	7.08	< 0.001	1.40
AQ – Physical aggression	17.43 (5.29)	13.96 (3.54)	3.90	0.002	0.77
AQ – Verbal aggression	12.69 (4.11)	10.49 (2.49)	3.26	0.016	0.65
AQ – Anger	21.16 (4.43)	16.53 (4.04)	5.51	< 0.001	1.09
AQ – Hostility	26.76 (5.90)	19.76 (6.42)	5.73	< 0.001	1.14
**Interpersonal Problems (IIP-32)**	2.82 (0.43)	2.52 (0.45)	3.35	0.013	0.66

*BPD* Borderline personality disorder, *CC* Clinical controls, *BSL-23* Short version of the Borderline Symptom List, *SCID-5-PD* Structured Clinical Interview for DSM-5 Personality Disorders, *F-IAT* Forgiveness Implicit Association Test, *TRIM* Transgression-Related Interpersonal Motivations Inventory, *TTF* Tendency to Forgive Scale, *AQ* Aggression Questionnaire, *IIP-32* Inventory of Interpersonal Problems-32

### Group differences in forgiveness

Welch’s *t*-tests with Bonferroni-Holm correction revealed no significant differences between the BPD and CC groups across all forgiveness measures (Table 1). While the CC group showed slightly higher scores on F-IAT, TTF, and TRIM – Benevolence, and lower scores on TRIM – Revenge and TRIM – Avoidance, these differences did not reach statistical significance after correcting for multiple comparisons. Overall, the findings suggest that no robust group differences in forgiveness emerged.

### Associations between forgiveness and BPD indicators

Correlational analyses revealed no significant associations between forgiveness measures and BPD indicators (BSL-23 and SCID-5-PD; Table 2). While some correlation coefficients suggested weak relationships, none reached statistical significance. Correlation matrices for all variables in both samples are presented in the Supplement (Figures S2 and S3).

**Table 2:** Intercorrelations between DIF (indirect, direct), BPD indicators and clinical variables in BPD (*N* = 51)

	F-IAT	TRIM			TTF
		Revenge	Avoidance	Benevolence	
Borderline pathology (BSL-23)	0.16	− 0.20	− 0.19	0.18	0.07
No. of BPD criteria (SCID-5-PD)	0.07	0	− 0.06	0.01	− 0.15
Aggression (AQ)	0.05	0.30*	0.14	− 0.07	− 0.40**
AQ – Physical aggression	− 0.04	0.15	− 0.03	− 0.10	− 0.29*
AQ – Verbal aggression	− 0.14	0.27	0.12	− 0.04	− 0.18
AQ – Anger	0.06	0.31*	0.14	−04	− 0.21
AQ – Hostility	0.21	0.13	0.17	− 0.07	− 0.38**

*BPD* Borderline personality disorder, *SCL-K-9* Symptom check list – 9 short version, *BSL-23* Short version of the Borderline Symptom List, *SCID-5-PD* Structured Clinical Interview for DSM-5 Personality Disorders, *F-IAT* Forgiveness Implicit Association Test, *TRIM* Transgression-Related Interpersonal Motivations Inventory, *TTF* Tendency to Forgive Scale, *AQ* Aggression Questionnaire

*p <.05, **p <.01

### Associations between forgiveness and clinical variables

Within the BPD sample, higher TRIM – Revenge scores were associated with greater aggression, particularly anger, while TTF showed negative associations with overall aggression, physical aggression, and hostility, suggesting that greater trait forgiveness was linked to lower aggression levels. No associations were found for F-IAT or TRIM – Avoidance and Benevolence.

**Fig. 1:**
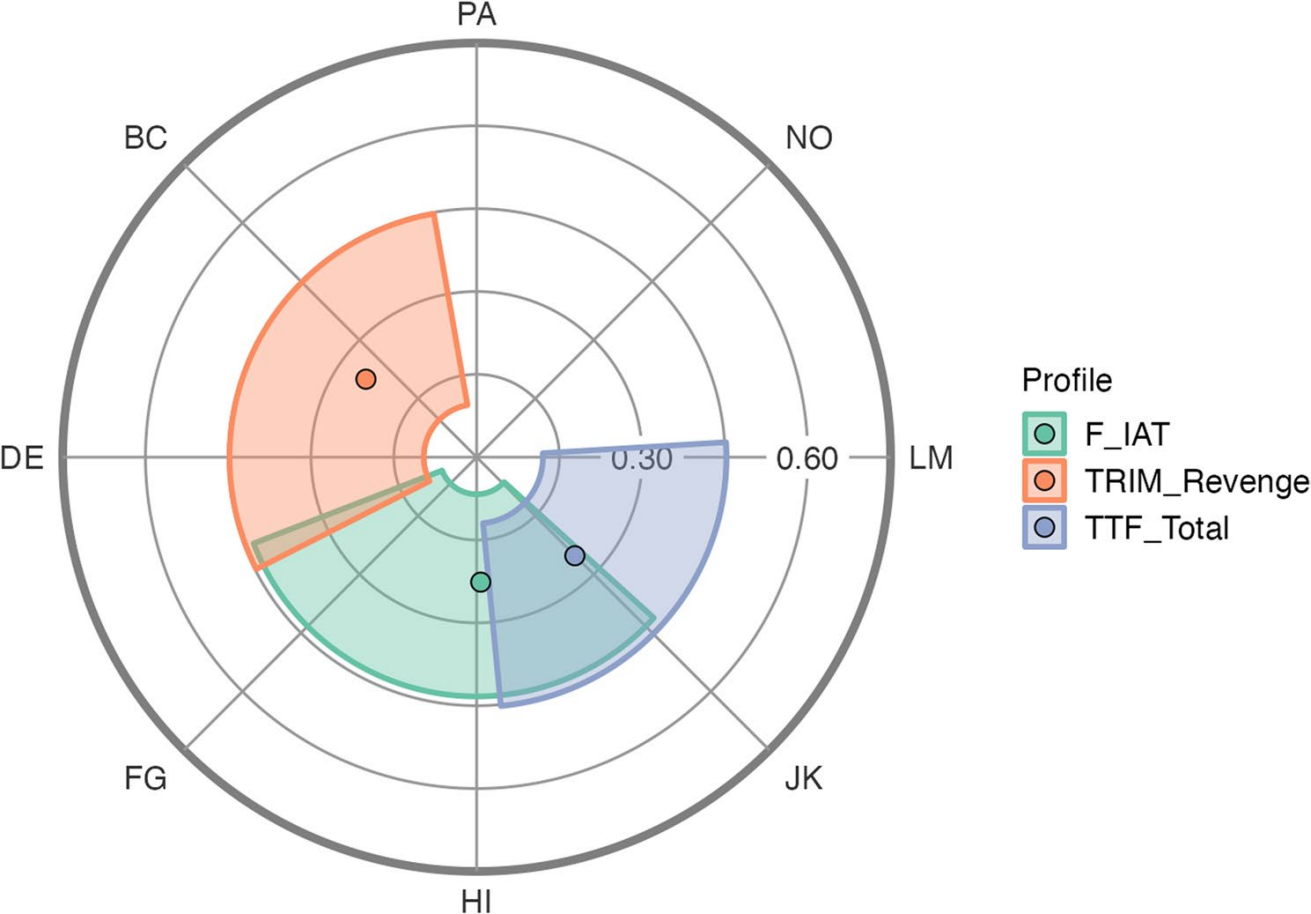
Projection of forgiveness outcomes onto the interpersonal circumplex model in the BPD sample (*N* = 51) Note. Forgiveness outcomes were projected using the Structural Summary Method (SSM) with bootstrapped 95% confidence intervals. Only associations with sufficient prototypicality (R² ≥ 0.70) are displayed; IIP-32 = Inventory of Interpersonal Problems-32; PA = Domineering; NO = Intrusive; LM = Self-sacrificing; JK = Exploitable; HI = Nonassertive; FG = Socially inhibited; DE = Cold; BC = Vindictive; DIF = Dispositional Interpersonal Forgiveness; F-IAT = Forgiveness Implicit Association Test; TRIM = Transgression-Related Interpersonal Motivations Inventory; TTF = Tendency to Forgive Scale

To further examine the interpersonal relevance of forgiveness, outcomes were projected onto the IIP-32 circumplex. Only measures with prototypical correlation profiles (*R*² ≥ 0.70) are displayed in Fig. 1. The F-IAT showed moderate elevation (e = 0.114, 95% CI [0.001, 0.213]) and moderate specificity (α = 0.226, 95% CI [0.064, 0.428]), with an acceptable model fit (*R*² = 0.841). This indicates that the F-IAT scores were moderately associated with interpersonal dysfunction, showed a moderately distinct interpersonal pattern, and were well represented within the circumplex structure.

Displacement indicated non-assertive interpersonal tendencies (δ = 271.87°, 95% CI [193.17°, 316.26°]). The TRIM – Revenge scale exhibited moderate elevation (e = 0.104, 95% CI [−0.003, 0.211]) and moderate specificity (α = 0.245, 95% CI [0.092, 0.438]), with an adequate model fit (R² = 0.804). This indicates moderate associations with interpersonal dysfunction, a moderately differentiated interpersonal pattern, and scores that were adequately captured within the circumplex structure. Displacement suggested a cold and competitive interpersonal pattern (δ = 144.85°, 95% CI [102.84°, 208.84°]). The TRIM – Avoidance scale showed low elevation (e = 0.074, 95% CI [−0.060, 0.206]) and weak specificity (α = 0.121, 95% CI [0.032, 0.330]), with moderate model fit (*R*² = 0.607), making displacement uninterpretable. The TRIM – Benevolence scale exhibited negligible interpersonal relevance, with very low elevation (e = −0.008, 95% CI [−0.135, 0.122]) and minimal specificity (α = 0.022, 95% CI [0.021, 0.262]). The poor model fit (*R*² = 0.032) ruled out displacement interpretation. The TTF total score showed the strongest interpersonal alignment, with moderate elevation (e = −0.098, 95% CI [−0.221, 0.015]) and high specificity (α = 0.252, 95% CI [0.127, 0.444]). The high model fit (*R*² = 0.853) allowed for displacement interpretation, indicating an association with warm and non-dominant tendencies (δ = 314.92°, 95% CI [275.70°, 8.00°]).

## Discussion

By jointly assessing explicit and implicit forgiveness self-concepts, this study provides a nuanced perspective on how forgiveness may manifest in women with BPD. While previous research has suggested diminished forgiveness tendencies in individuals with BPD [27, 29], our study offers a more differentiated perspective. Using both direct (TRIM, TTF) and indirect (F-IAT) measures of forgiveness, we observed largely comparable forgiveness tendencies between women with BPD and a matched clinical control (CC) group. Similarly, associations between forgiveness measures and BPD symptomatology as assessed by the BSL-23 and the number of SCID-5 BPD criteria were small and non-significant. These findings stand in contrast to earlier work and may reflect the high internal variability and conceptual complexity of forgiveness, a socially desirable and context-sensitive construct, which may limit its diagnostic sensitivity in distinguishing between clinical subgroups. Rather than serving as a diagnostic marker, forgiveness may be more meaningfully considered in relation to interpersonal functioning and relational themes in BPD. Its potential relevance in clinical contexts warrants further investigation.

Alternatively, the absence of significant effects could be due to sample-specific or methodological factors. First, it is possible that the matched clinical control group, likely also burdened with high levels of interpersonal stress and trauma, showed similar forgiveness tendencies, thereby minimizing between-group contrasts. Second, one alternative explanation for the lack of convergence may lie in underlying features of BPD, such as identity diffusion and emotional ambivalence [57], which could contribute to inconsistent self-representations and thus affect both direct and indirect forgiveness assessments. Finally, the absence of differences could indicate that forgiveness is not a stable trait-like deficit in BPD but rather context-dependent and dynamically influenced by interpersonal and situational factors.

Nonetheless, several differential patterns of association emerged within the BPD group. Explicit measures of forgiveness – particularly the TTF and TRIM-Revenge – showed theoretically consistent associations with clinical characteristics within the BPD group. Specifically, a higher tendency to forgive was associated with lower aggression, particularly hostility and physical aggression, while stronger revenge motivations (TRIM-Revenge) correlated positively with anger and overall aggression. These findings replicate and extend previous work linking forgiveness to maladaptive emotional and behavioral outcomes [10, 47], and highlight forgiveness as a potentially relevant correlate of aggressive tendencies in BPD. Notably, the F-IAT, our implicit measure of forgiveness, did not show significant associations with aggression, borderline pathology, or self-reported forgiveness. While this may be seen as a methodological limitation, it is also consistent with dual-process models [58, 59], which distinguish between automatic and reflective cognitive systems and suggest that implicit and explicit measures often show only limited convergence [60]. From this perspective, our findings may indicate that implicit and explicit forgiveness tap into related but distinct aspects of self-related processing. In the context of BPD, an alternative explanation may relate to the roles of emotional dysregulation and identity instability, both of which frequently affect self-representation in this population [9]. In these cases, discrepancies between explicit self-reports and implicit evaluative tendencies may indicate underlying tensions in the self-concept. One possible manifestation of this divergence refers to the phenomenon of “hollow forgiveness,” in which individuals consciously endorse forgiving attitudes while unresolved negative evaluations persist implicitly [35]. These processes warrant further investigation.

Further insight into the interpersonal relevance of forgiveness emerged from circumplex modeling. The Structural Summary Method revealed that the forgiveness measures varied in their alignment with interpersonal styles. The TTF showed high interpersonal specificity, aligning with warm, non-dominant traits, while TRIM-Revenge was associated with a cold, competitive interpersonal style – consistent with a withdrawal-oriented, retaliatory stance. The F-IAT, though only modestly elevated, aligned with nonassertive interpersonal tendencies, potentially reflecting the passive, internalized nature of implicit forgiveness representations. Notably, the TRIM-Benevolence scale did not meaningfully map onto interpersonal styles, suggesting limited interpersonal salience within this sample. These patterns suggest that specific dimensions of forgiveness are differentially embedded in the interpersonal functioning of individuals with BPD. Taken together, this underscores how forgiveness is not a unitary construct but interacts with interpersonal functioning in nuanced ways. Although forgiveness may not currently serve as a direct focus in clinical practice, longitudinal research suggests it holds clinical relevance: recovered individuals with BPD report significantly higher levels of forgiveness and acceptance over time than non-recovered patients [11], indicating its potential as a meaningful therapeutic target. We regard this study as a foundational step toward a more differentiated understanding of forgiveness in BPD and hope it will encourage future research to investigate how forgiveness interacts with broader social-cognitive processes and affective dynamics in this population.

Before concluding, some limitations of the present study should be acknowledged: First, we only compared forgiveness between individuals with BPD and a CC without any personality disorder. As we did not include a comparison group of individuals with other personality disorders (PDs), it remains unclear whether our findings are specific to BPD, as suggested by Zanarini and colleagues [29], or reflect broader patterns of personality pathology. In addition, since our sample consisted exclusively of female participants, the generalizability of the findings remains to be established and should be examined in future studies including more gender-diverse samples. Prior research has reported gender differences in forgiveness tendencies [38], which may also extend to clinical populations. Second, no a priori power analysis was conducted, primarily due to the limited availability of previous studies on forgiveness in BPD. Although our sample size of 51 participants per group meets general standards for medium-sized effects, it may be not sufficiently powered to detect more subtle or interaction effects, especially in exploratory analyses involving implicit measures. Accordingly, null findings – particularly those concerning group differences or weak associations – should be interpreted with caution. Future studies should determine sample sizes based on power analyses tailored to forgiveness-related constructs to ensure more robust conclusions. To this end, the Forgiveness Implicit Association Test (F-IAT) may offer a complementary, though still novel, tool for capturing forgiveness-related processes [37]. Furthermore, due to the cross-sectional nature of our design, no causal inferences can be drawn regarding the relationship between forgiveness and clinical features of BPD. It remains to be seen whether low forgiveness contributes to increased aggression and interpersonal dysfunction in BPD, or whether these clinical features, in turn, impair an individual’s dispositional tendency to forgive. Longitudinal designs are needed to examine the temporal dynamics of forgiveness and its relevance for symptom trajectories or treatment outcomes in BPD. Our study focused exclusively on forgiveness tendencies and did not differentiate between different targets or types of forgiveness (e.g., forgiveness of close others vs. distant acquaintances, or decisional vs. emotional forgiveness). At last, we did not assess the potential impact of trauma histories, such as CM, on forgiveness processes –despite prior evidence linking trauma to impaired forgiveness [19, 20]. Future research should aim to include more fine-grained assessments of forgiveness and, incorporate relevant contextual variables (e.g., trauma, attachment, social-cognitive impairments) and, consider the role of self-forgiveness versus interpersonal forgiveness in BPD populations.

In conclusion, our findings offer a nuanced picture of forgiveness in BPD. Although implicit and explicit measures of forgiveness showed limited overlap, both were systematically related to aggression and interpersonal problems within the BPD group. However, overall forgiveness levels did not differ from those of matched clinical controls. This lack of group differences suggests that forgiveness is a context-dependent, dynamic process in BPD rather than a stable, deficit-like trait.

These findings encourage a multidimensional approach to studying forgiveness in BPD and reinforce the value of integrating both direct and indirect methods to capture the complexity of self-related processes in personality pathology. While primarily relevant to clinical research, we believe the results can also inform clinical practice by shedding light on implicit dynamics that may underlie interpersonal difficulties and treatment challenges in individuals with BPD.

## Supplementary Information

Supplementary Material 1.

## Data Availability

The supplementary materials and R code have been made publicly available at the Open Science Framework and can be accessed at https://osf.io/rba48/. For ethical reasons, the data from this study are available only upon reasonable request from the last author.

## Abbreviations

BPD: Borderline Personality Disorder
CC: Clinical Controls
IAT: Implicit Association Test
APA: American Psychiatric Association
FFM: Five Factor Model of personality
DSM-5: Diagnostic and Statistical Manual of Mental Disorders 5^th^ Edition
CM: Childhood maltreatment
DBT: Dialectical behavior therapy
JARS: Journal Article Reporting Standards
