# Supplementary material for: Implicit and explicit self-concepts of forgiveness in women with borderline personality disorder
Source: Borderline Personal Disord Emot Dysregul. 2025 Sep 2;12:35. doi: 10.1186/s40479-025-00312-4 (PMC12406368; doi:10.1186/s40479-025-00312-4)

**Figure S1**. Flow of participants through each stage of the study


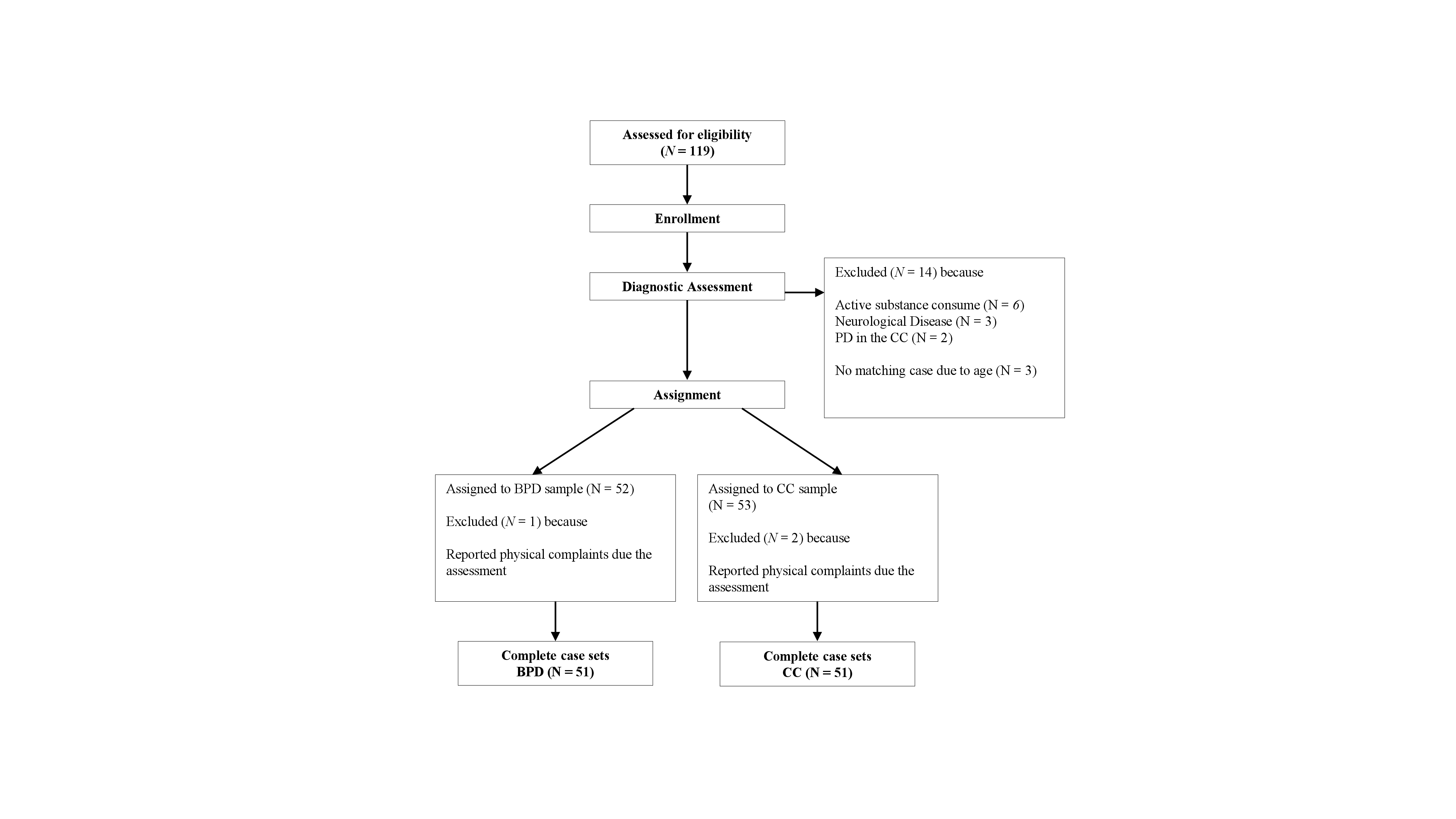


**Table S1** Frequency table for the Mini-DIPS diagnoses in the BPD (N = 51) and CC (N = 51) samples.

| Diagnoses | BPD (%) | CC (%) | χ² | df | *p* |
| --- | --- | --- | --- | --- | --- |
| Anxiety Disorder | 31 (61%) | 20 (39%) | 3.92 | 1 | .048 |
| Panic Disorder | 19 (37%) | 7 (14%) | 6.25 | 1 | .012 |
| Agoraphobia | 19 (37%) | 10 (20%) | 3.08 | 1 | .079 |
| Social Anxiety Disorder | 10 (20%) | 2 (4%) | 4.63 | 1 | .031 |
| Specific Phobia | 11 (22%) | 7 (14%) | 0.61 | 1 | .436 |
| Generalized Anxiety Disorder | 10 (20%) | 9 (18%) | 0 | 1 | 1 |
| Major Depression | 42 (82%) | 46 (90%) | 0.75 | 1 | .388 |
| Obsessive-Compulsive Disorder | 5 (10%) | 3 (6%) | 0.14 | 1 | .712 |
| PTSD | 18 (35%) | 3 (6%) | 11.75 | 1 | .001 |
| Eating Disorder | 13 (25%) | 5 (10%) | 3.31 | 1 | .069 |
| Somatoform Disorder | 9 (18%) | 5 (10%) | 0.75 | 1 | .388 |

Note. Mini-DIPS= Short form of the Diagnostic Interview for Mental Disorders Open Access

**Figure S2** Intercorrelations between BPD indicators, forgiveness, and all clinical measures in the BPD sample (N=51)


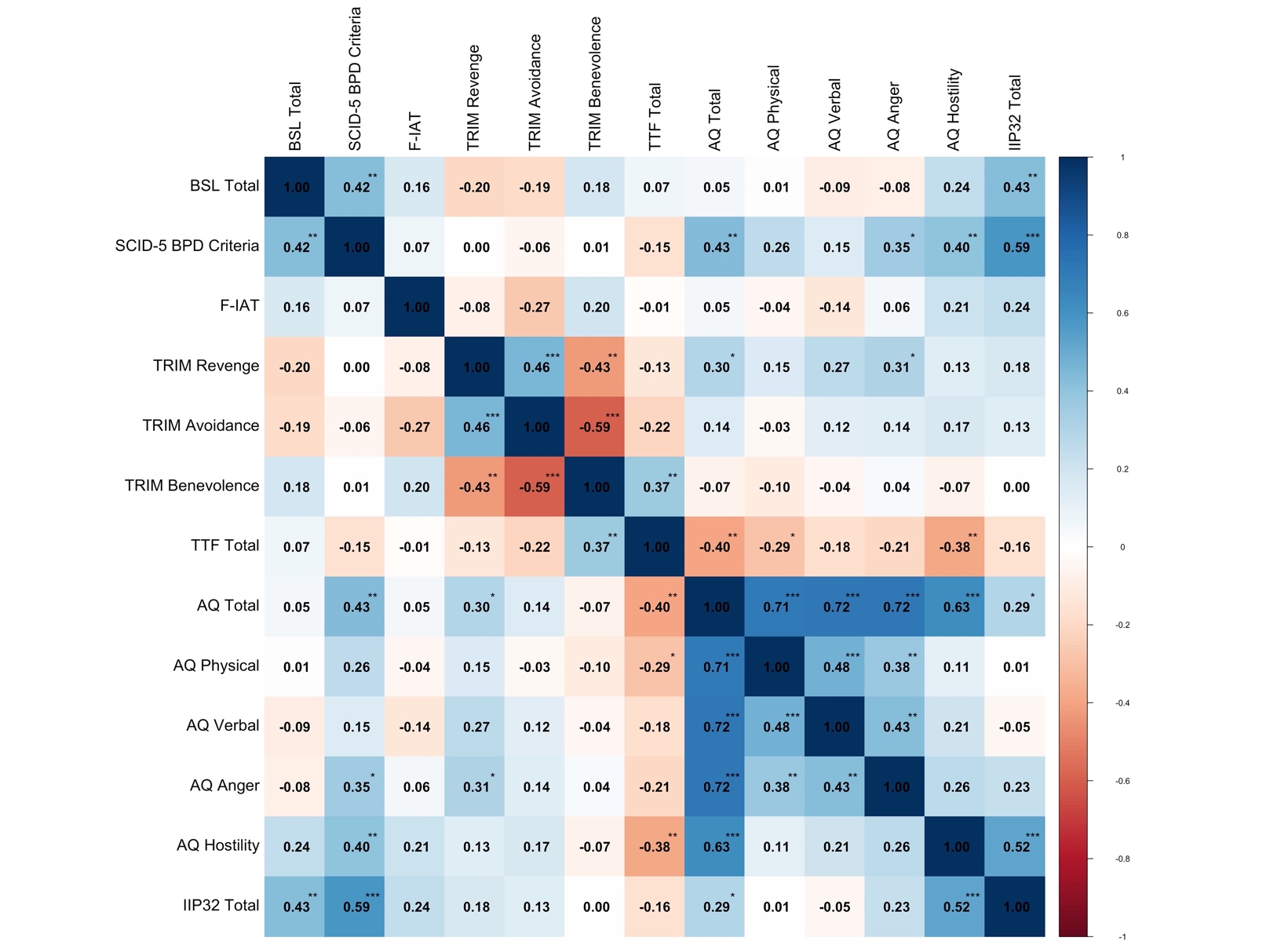


**Figure S3** Intercorrelations between BPD indicators, forgiveness, and all clinical measures in the CC sample (N=51)


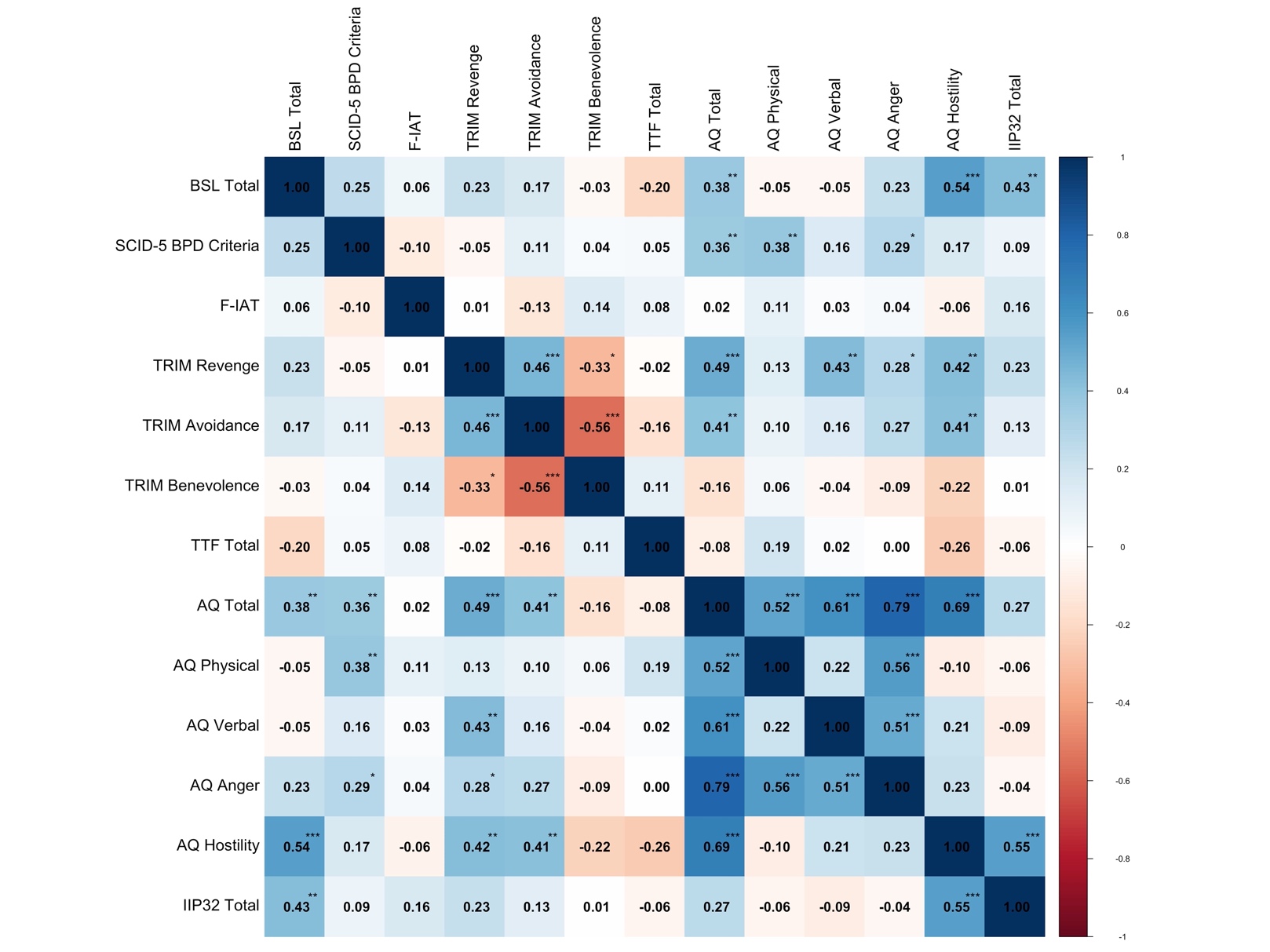

Supplement: Supplementary file 1 — Supplementary Material 1. [file 40479_2025_312_MOESM1_ESM.docx]
